# Screen time, impulsivity, neuropsychological functions and their relationship to growth in adolescent attention-deficit/hyperactivity disorder symptoms

**DOI:** 10.1038/s41598-023-44105-7

**Published:** 2023-10-23

**Authors:** Jasmina Wallace, Elroy Boers, Julien Ouellet, Mohammad H. Afzali, Patricia Conrod

**Affiliations:** 1 CHU Sainte-Justine Research Center, Montreal, QC Canada; 2 Department of Psychiatry, University of Montreal, Montreal, QC Canada

**Keywords:** Risk factors, Neuroscience, Medical research

## Abstract

Previous longitudinal studies found significant associations between screen time and increase in attention-deficit/hyperactivity disorder (ADHD) symptoms, but the mechanisms mediating this association remain understudied. Thus, we used data from a 5-year population-based longitudinal cohort of nearly 4000 Canadian high school students, modeled using multivariate multilevel mediation, to investigate the association of screen time (i.e., social media, television, video games, computer use) with ADHD symptoms via different potential behavioral and neuropsychological mediators (i.e. impulsivity, response inhibition, working memory). We studied direct and indirect between-person, concurrent within-person, and lagged-within-person effects of screens on ADHD symptoms. Results showed that increases in screen time in a given year were associated with an exacerbation of ADHD symptoms within that same year (within-person association), over and above potential common vulnerability (between-person association). Impulsivity proved to be the most robust mediator in the association of screen time with ADHD symptoms at both between and within-person levels. Only social media use displayed a significant lagged-within-person association with ADHD symptoms mediated by impulsivity, indicating an enduring influence on behavior, which was further shown to be mediated by chained changes in response inhibition on a Go/No-Go task. These findings provide clinical implications of screen time and should be an important focus in the management and prevention of ADHD symptoms among adolescents.

## Introduction

Attention-deficit/hyperactivity disorder (ADHD) is a behavioral disorder characterized by symptoms of inattention and/or impulsivity and hyperactivity, which can affect many aspects of behavior and performance among youth, both at school and at home^1^. ADHD during adolescence has been associated not only with neuropsychological functions, but also with significant academic, psychosocial, emotional, and cognitive impairment. For instance, it has been linked to poor social relationships, low self-esteem, deviant behavior, and substance use and abuse^2^.

In 2016, an estimated 6.1 million North-American children 2–17 years of age (9.4%) had received an ADHD diagnosis, of which the highest rates of ADHD were found among adolescents aged 12–17 years^3,4^. Furthermore, the U.S. national prevalence of ADHD was shown to increase by 12.6% over the past decade (from 8.47 to 9.54% from 2009–2011 to 2015–2017)^4^. It has been estimated that U.S. healthcare expenditures for ADHD total $23 billion^5^ and that the annual costs including healthcare, education, and reduced family productivity associated with youth ADHD in the U.S. have been estimated to range from $38 to $72 billion^6^.

Considering that ADHD risk is linked to both genetic and non-genetic environmental risk factors, identifying potential environmental factors that might explain epidemiologic shifts in ADHD severity or prevalence might help to inform more comprehensive treatment and public health strategies to promote youth mental health^7^. One of these environmental risk factors might be the rise of continuous expansion of the digital media landscape, which can be considered one of the most dramatic cultural changes that has occurred in the past decade. The ubiquity of internet access, social media platforms, online video gaming, and of the wide variety of mobile digital devices result in near-constant exposure to virtual information, which may interfere with adolescent neurodevelopment processes^8,9^ and make them increasingly digitally dependent showing behavior disorders^10–14^.

It has been suggested that North-American youth spend an average of 6–9 h interacting with digital devices, and it must be pointed out that many young people often use more than one screen medium simultaneously, increasing their total exposure to digital devices^15^. Moreover, the recent outbreak of the COVID-19 pandemic has resulted in a substantial increase in screen time among adolescents^16–18^. Finally, it is noteworthy that many digital platforms are designed to tap natural human attentional, impulsive, and reward processes, which in turn leads to habit formation and repeated use of the platform^13,19–23^.

Research on the impact of digital screen time on adolescent health is still in its infancy and the neurodevelopmental consequences of exposure to such digital platforms are not yet fully understood. This is particularly relevant to ADHD symptoms, as impulsivity, reward processing, and attention/concentration are key features that drive ADHD behavioral impairments^24,25^. In particular, deficits in working memory are typical among individuals with ADHD^26^, and a meta-analysis found significantly more and more severe verbal and visual-spatial working memory impairments in adolescents with ADHD compared to non-ADHD adolescents^27^. Previous works also suggested that those diagnosed with ADHD have been shown to differ from those without a diagnosis on a variety of tests of inhibitory control, such as, go/no-go tasks^28–31^ and continuous performance tasks^32,33^.

Findings from emerging research suggest that excessive usage of digital media in adolescents may affect brain functioning and cognitive development, including impaired attention and impaired memory processing, as well as impairment in impulse regulation and reward processing^13,34,35^. More specifically for social media, heavy Facebook users had worse short-term memory than light users^36^ and problematic use of social networking sites has been associated with attention problems^37^. Moreover, previous studies have shown that total television and video game exposure were related to future attention problems, controlling for earlier attention^38,39^. Longitudinal studies also showed significant, though modest, associations between modern digital media use (e.g. checking social media, texting, posting, etc.) and symptoms of ADHD^40^, as well as long-term effects of media multitasking on attention problems for adolescents aged 11 and 13 years old^41^.

Although the above-presented findings of previous works suggest an important relationship between digital media use and ADHD symptom severity, little research directly tested mediation hypotheses^42^, and even fewer have attempted to address these hypotheses by investigating potential pathways through the effect of digital media use on neuropsychological development in adolescence. As previously suggested^42^, mediating variables could shine a light on the underlying mechanism between screen time and its consequences, thereby providing further evidence of a causal relationship^43^. Furthermore, from a methodological perspective, ignoring mediating variables could lead to an underestimation of effect sizes in empirical research, and subsequent meta-analyses^44^. Hence, to obtain a true understanding of the association of digital media use and ADHD symptoms through direct and indirect effects, there is a need for studies that measure adolescents’ responses to different digital media over time, while taking into account mediating factors. The empirical findings and insights derived from such studies may prove pivotal in designing remedial and preventive interventions to assist in the management of ADHD-related behaviors among adolescents. To address this gap in the literature, potential mediating factors of interest could be impulsivity and neuropsychological functions, which are not only identified as core behavioral and cognitive features of ADHD^25,45^, but also seem to be affected by screen time, as reviewed above. Demonstrating chained mediation of cognitive processes in the link between screentime and mental health would contribute to a growing literature on potential causal effects of screentime on child brain health.

In this study, we used multivariate multilevel linear models (MLMs) to analyze longitudinal data to test causal hypotheses on the role of screen time (i.e., social media use, television viewing, video gaming, and computer use) in the growth of ADHD symptoms across adolescence, by: (i) first modeling, and controlling for potential common vulnerability to high levels of digital media use, impulsivity, neuropsychological functioning, and ADHD symptoms (between-person effects); (ii) modeling how changes in impulsivity and neuropsychological functioning co-vary directly with an increase in digital media use in the same year and the following year (concurrent within-person effects and lagged within-person effects, respectively); (iii) modeling how impulsivity and neuropsychological functions mediate indirect associations of digital media and ADHD symptoms at between-, within- and lagged-within person levels; (iv) and also compared the four types of screen time as predictors of ADHD symptoms and neuropsychological functioning to explore if certain types of digital media are particularly linked to cognitive and mental health outcomes.

We designed a multilevel analytic strategy in order to be able to differentiate between-person variation from within-person variation. The former reflects the time-invariant degree to which individuals differ in their average scores from others in the sample, in other words, the set point of an individual compared with their peers. The latter effect represents the time-varying degree to which individuals deviate from their own average scores when tested in successive years through repeated measures, representing the displacement from the set point at a given time point. Therefore, our longitudinal study allowed disaggregating inter-individual and intra-individual differences in the association of screen time and ADHD symptoms.

This study also applies the same strategy to investigating neurocognitive and behavioral mediators of this relationship to confirm its validity, by including objectively measured (task-based) response inhibition and working memory performance, as well as self-reported impulsivity, annually for five consecutive years. We will uniquely investigate temporal precedence of neurocognitive mediators in this relationship, in accordance with current etiologic theories of ADHD placing response inhibition, working memory and trait impulsivity at the core of this complex brain disorder^25,45^.

## Results

### Descriptive statistics

The sample included 3779 adolescents (1858 girls [49%]; mean [SD] age, 12.8 [0.5] years) who consented and completed the first survey of this 5-year longitudinal study.

The descriptive statistics of each variable and the frequency distribution for the different types of screen time over 5 years are available in Tables S1 and S2, respectively, as Supplementary Information.

As shown by the frequency distribution for the different types of screen time, we reported an increase in missing data over the course of the study (0.30% of missing data relative to all data at year 1; 32% of missing data relative to all data at year 5).

Over the 5-year period, there were reported substantial increases in minimum time spent using social media (year 1 mean[SD], 42[63] minutes; year 5 mean[SD], 70[66] minutes) and slight increases in time spent viewing television (year 1 mean[SD], 66[63] minutes; year 5 mean[SD], 72[66] minutes) and using the computer (year 1 mean[SD], 25[50] minutes; year 5 mean[SD], 26[50] minutes), while there were reported decreases in video gaming (year 1 mean[SD], 59[69] minutes; year 5 mean[SD], 55[72] minutes).

Scores related to ADHD symptoms increased across the 5-year study (year 1 mean[SD], 3.93[2.31]; year 5 mean[SD], 4.06[2.36]). Impulsivity reported stable scores across the five survey waves (year 1 mean[SD], 11.88[2.84] points; year 5 mean[SD], 11.31[2.84] pints). Concerning neuropsychological function variables, both response inhibition errors (year 1 mean[SD], 31.28[17.62]; year 5 mean[SD], 16.16[15.02]) and working memory errors (year 1 mean[SD], 16.05[10.19]; year 5 mean[SD], 8.89[8.40]) decreased from year-to-year.

Participants reported a mean[SD] socio-economic status score of 2.94[1.08].

### The direct associations of screen time and ADHD symptoms

**Figure 1:**
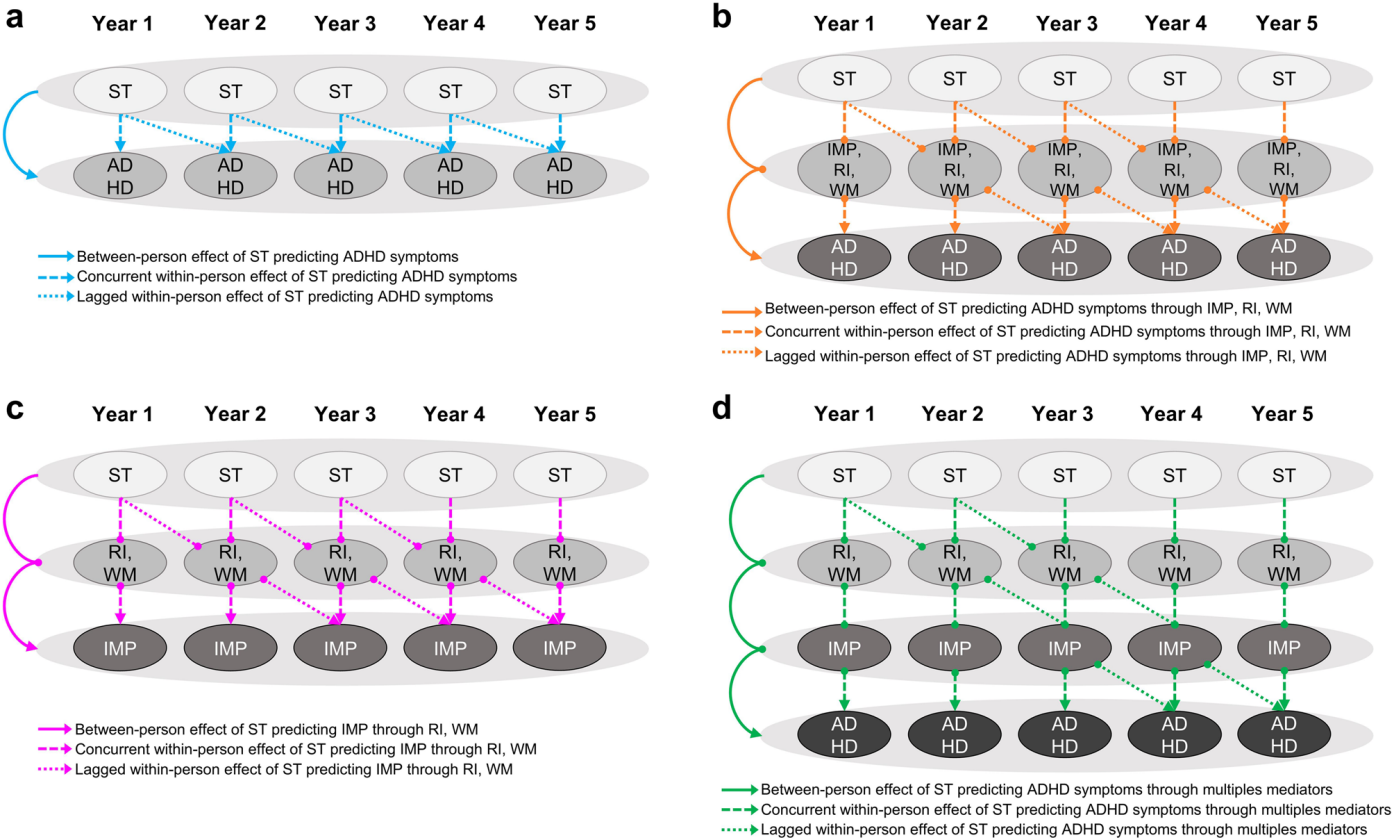
Visualization of statistical mediation multilevel models. Mediation multilevel models were applied to assess the associations of screen time and attention-deficit/hyperactivity disorder (ADHD) symptoms (**a**), the associations of screen time and ADHD symptoms through impulsivity and neuropsychological functions (**b**), the associations of screen time and impulsivity through neuropsychological functions (**c**), and the associations of screen time and ADHD symptoms through multiple mediators (**d**). ADHD, attention-deficit/hyperactivity disorder symptoms; IMP, impulsivity; RI, response inhibition; ST, screen time; WM, working memory; Year 1: assessment with first survey wave at 7th grade, and so on.

All results of MLM analysis indicating the direct associations of screen time and ADHD symptoms among adolescents (according to the model schematized in Fig. 1a) are shown in Table 1. The results showed a statistically significant between-person effect of social media use, television viewing, and video gaming on ADHD symptoms. Results also indicated a statistically significant concurrent within-person effect of social media use, television viewing, and video gaming on ADHD symptoms. Computer use showed no statistical significant association with ADHD symptoms at between and within levels. Since computer use did not show significant direct associations with ADHD symptoms, we did not include it in further statistical analysis. No further statistically significant lagged-within-person effect was found for screen time on ADHD symptoms.

**Table 1:** Estimated parameters for multilevel models, as schematized in Fig. 1a, assessing the direct effect of social media use, television viewing, video gaming, and computer use on attention-deficit/hyperactivity disorder (ADHD) symptoms.

Predictors	Estimate	Std. Er	Pr( >|t|)	95% CI
Social media use				
Intercept	− 0.605	0.064	0.000	− 0.722, − 0.490
Time	0.169	0.005	0.000	0.158, 0.178
Sex	0.678	0.030	0.000	0.615, 0.731
Socio-economic status	0.071	0.008	0.000	0.052, 0.085
Between-person	**0.481**	**0.051**	**0.000**	**0.350, 0.564**
Within-person	**0.134**	**0.018**	**0.000**	**0.098, 0.169**
Lagged-within-person	0.012	0.019	0.245	− 0.031, 0.047
Television viewing				
Intercept	1.100	0.071	0.000	0.960, 1.231
Time	0.029	0.004	0.000	0.022, 0.038
Sex	0.283	0.031	0.000	0.214, 0.329
Socio-economic status	− 0.003	0.009	0.370	− 0.022, 0.015
Between-person	**0.122**	**0.052**	**0.005**	**0.020, 0.232**
Within-person	**0.056**	**0.016**	**0.000**	**0.023, 0.084**
Lagged-within-person	− 0.004	0.018	0.390	− 0.049, 0.026
Video gaming				
Intercept	2.453	0.073	0.000	2.313, 2.581
Time	− 0.026	0.004	0.000	− 0.034, − 0.018
Sex	− 0.714	0.034	0.000	− 0.774, − 0.642
SES	0.009	0.009	0.145	− 0.008, 0.026
Between-person	**0.218**	**0.052**	**0.000**	**0.114, 0.315**
Within-person	**0.071**	**0.013**	**0.000**	**0.047, 0.098**
Lagged-within-person	0.029	0.018	0.035	− 0.004, 0.064
Computer use				
Intercept	0.890	0.051	0.000	0.791, 1.000
Time	0.010	0.005	0.010	0.001, 0.021
Sex	− 0.150	0.023	0.000	− 0.195, − 0.107
Socio-economic status	− 0.017	0.006	0.005	− 0.030, − .007
Between-person	− 0.059	0.080	0.175	− 0.227, 0.085
Within-person	0.018	0.016	0.170	− 0.021, 0.045
Lagged-within-person	0.023	0.022	0.180	− 0.019, 0.066

The significant between- and within-person associations are highlighted in boldface (*p* < 0.050, one-tailed *p*-value).

### The indirect associations of screen time and ADHD symptoms through impulsivity and neuropsychological functions

Results of MLM analysis assessing the association of social media use, television viewing and video gaming on ADHD symptoms in adolescents through impulsivity and neuropsychological functions (according to the model schematized in Fig. 1b) are shown in Tables 2, 3 and 4, respectively.

**Table 2:** Estimated parameters for mediation multilevel models, as schematized in Fig. 1b, assessing impulsivity, response inhibition, and working memory as mediators of the temporal association of social media use and attention-deficit/hyperactivity disorder (ADHD) symptoms.

Predictors	Estimate	Std. Er	Pr( >|t|)	95% CI
Social media predicting ADHD symptoms through impulsivity				
Intercept (ADHD)	− 3.267	0.206	0.000	− 3.738, − 2.943
Intercept (impulsivity)	12.509	0.161	0.000	12.197, 12.789
Social media on ADHD between-person	0.108	0.038	0.005	0.029, 0.181
Social media on ADHD within-person	0.101	0.014	0.000	0.075, 0.131
Social media on ADHD lagged-within-person	− 0.005	0.018	0.360	− 0.040, 0.032
Social media on impulsivity between-person	0.830	0.047	0.000	0.737, 0.918
Social media on impulsivity within-person	0.225	0.021	0.000	0.186, 0.264
Social media on impulsivity lagged-within-person	0.071	0.026	0.000	0.014, 0.116
Impulsivity on ADHD between-person	0.586	0.014	0.000	0.559, 0.618
Impulsivity on ADHD within-person	0.255	0.007	0.000	0.242, 0.270
Impulsivity on ADHD lagged-within-person	0.030	0.008	0.000	0.013, 0.045
Mediation between-person	**0.484**	**0.030**	**0.000**	**0.430, 0.546**
Mediation within-person	**0.057**	**0.006**	**0.000**	**0.047, 0.068**
Mediation lagged-within-person	**0.002**	**0.001**	**0.000**	0.000, 0.004*
Social media predicting ADHD symptoms through response inhibition				
Intercept (ADHD)	3.597	0.216	0.000	3.134, 3.969
Intercept (response inhibition)	35.555	0.986	0.000	33.654, 37.563
Social media on ADHD between-person	0.575	0.053	0.000	0.478, 0.678
Social media on ADHD within-person	0.131	0.020	0.000	0.086, 0.167
Social media on ADHD lagged-within-person	− 0.010	0.023	0.285	− 0.063, 0.030
Social media on response inhibition between-person	2.574	0.328	0.000	1.972, 3.261
Social media on response inhibition within-person	0.440	0.179	0.010	0.075, 0.725
Social media on response inhibition lagged-within-person	0.250	0.230	0.125	− 0.181, 0.689
Response inhibition on ADHD between-person	0.011	0.004	0.000	0.003, 0.018
Response inhibition on ADHD within-person	0.001	0.001	0.260	− 0.001, 0.003
Response inhibition on ADHD lagged-within-person	0.000	0.001	0.400	− 0.003, 0.003
Mediation between-person	**0.029**	**0.011**	**0.000**	**0.007, 0.050**
Mediation within-person	0.000	0.001	0.270	− 0.001, 0.002
Mediation lagged-within-person	0.000	0.001	0.465	− 0.001, 0.001
Social media predicting ADHD symptoms through working memory				
Intercept (ADHD)	3.863	0.189	0.000	3.486, 4.250
Intercept (working memory)	19.850	0.503	0.000	18.874, 20.863
Social media on ADHD between-person	0.580	0.045	0.000	0.490, 0.669
Social media on ADHD within-person	0.155	0.016	0.000	0.121, 0.187
Social media on ADHD lagged-within-person	0.023	0.020	0.143	− 0.019, 0.058
Social media on working memory between-person	1.498	0.154	0.000	1.184, 1.774
Social media on working memory within-person	0.192	0.073	0.000	0.049, 0.339
Social media on working memory lagged-within-person	0.092	0.100	0.177	− 0.101, 0.306
Working memory on ADHD between-person	0.008	0.006	0.117	− 0.004, 0.021
Working memory on ADHD within-person	0.004	0.002	0.073	− 0.001, 0.008
Working memory on ADHD lagged-within-person	0.005	0.002	0.007	0.001, 0.010
Mediation between-person	0.012	0.009	0.117	− 0.006, 0.031
Mediation within-person	0.001	0.001	0.073	0.000, 0.002
Mediation lagged-within-person	0.000	0.001	0.183	− 0.001, 0.002

The significant mediated between- and within-person associations are highlighted in boldface (*p* < 0.050, one-tailed *p*-value). *CI does not include/cross zero.

**Table 3:** Estimated parameters for mediation multilevel models, as schematized in Fig. 1b, assessing impulsivity, response inhibition, and working memory as mediators of the temporal association of television viewing and attention-deficit/hyperactivity disorder (ADHD) symptoms.

Predictors	Estimate	Std. Er	Pr( >|t|)	95% CI
Television viewing predicting ADHD symptoms through impulsivity				
Intercept (ADHD)	− 3.507	0.194	0.000	− 3.932, − 3.186
Intercept (impulsivity)	11.443	0.173	0.000	11.073, 11.744
Television viewing on ADHD between-person	0.081	0.041	0.045	− 0.006, 0.148
Television viewing on ADHD within-person	0.055	0.014	0.000	0.023, 0.085
Television viewing on ADHD lagged-within-person	− 0.001	0.019	0.440	− 0.040, 0.035
Television viewing on impulsivity between-person	0.525	0.054	0.000	0.425, 0.624
Television viewing on impulsivity within-person	0.092	0.021	0.000	0.050, 0.131
Television viewing on impulsivity lagged-within-person	− 0.032	0.026	0.085	− 0.089, 0.011
Impulsivity on ADHD between-person	0.595	0.013	0.000	0.566, 0.622
Impulsivity on ADHD within-person	0.259	0.007	0.000	0.246, 0.274
Impulsivity on ADHD lagged-within-person	0.031	0.008	0.000	0.014, 0.046
Mediation between-person	**0.313**	**0.034**	**0.000**	**0.257, 0.374**
Mediation within-person	**0.024**	**0.006**	**0.000**	**0.013, 0.034**
Mediation lagged-within-person	− 0.001	0.001	0.085	− 0.004, 0.000
Television viewing predicting ADHD symptoms through response inhibition				
Intercept (ADHD)	2.607	0.208	0.000	2.177, 2.994
Intercept (response inhibition)	32.871	1.032	0.000	31.088, 35.122
Television viewing on ADHD between-person	0.412	0.055	0.000	0.297, 0.523
Television viewing on ADHD within-person	0.052	0.019	0.005	0.011, 0.084
Television viewing on ADHD lagged-within-person	− 0.017	0.023	0.245	− 0.062, 0.025
Television viewing on response inhibition between-person	1.146	0.369	0.000	0.406, 1.886
Television viewing on response inhibition within-person	− 0.015	0.173	0.455	− 0.337, 0.265
Television viewing on response inhibition lagged-within-person	− 0.076	0.218	0.395	− 0.454, 0.348
Response inhibition on ADHD between-person	0.018	0.004	0.000	0.009, 0.025
Response inhibition on ADHD within-person	0.001	0.001	0.180	− 0.001, 0.003
Response inhibition on ADHD lagged-within-person	0.000	0.001	0.435	− 0.003, 0.003
Mediation between-person	**0.019**	**0.008**	**0.000**	**0.007, 0.040**
Mediation within-person	0.000	0.000	0.495	− 0.001, 0.000
Mediation lagged-within-person	0.000	0.000	0.490	0.000, 0.001
Television viewing predicting ADHD symptoms through working memory				
Intercept (ADHD)	2.897	0.187	0.000	2.527, 3.290
Intercept (working memory)	18.313	0.541	0.000	17.230, 19.349
Television viewing on ADHD between-person	0.360	0.049	0.000	0.261, 0.454
Television viewing on ADHD within-person	0.004	0.002	0.057	− 0.001, 0.008
Television viewing on ADHD lagged-within-person	− 0.004	0.019	0.423	− 0.042, 0.036
Television viewing on working memory between-person	0.601	0.181	0.000	0.235, 0.969
Television viewing on working memory within-person	0.152	0.074	0.027	− 0.005, 0.295
Television viewing on working memory lagged-within-person	− 0.016	0.096	0.437	− 0.198, 0.182
Working memory on ADHD between-person	0.020	0.006	0.010	0.008, 0.033
Working memory on ADHD within-person	0.004	0.002	0.057	− 0.001, 0.008
Working memory on ADHD lagged-within-person	0.005	0.002	0.010	0.001, 0.010
Mediation between-person	**0.011**	**0.005**	**0.010**	**0.004, 0.024**
Mediation within-person	0.001	0.000	0.077	0.000, 0.002
Mediation lagged-within-person	0.000	0.001	0.440	− 0.001, 0.001

The significant mediated between- and within-person associations are highlighted in boldface (*p* < 0.050, one-tailed *p*-value).

**Table 4:** Estimated parameters for mediation multilevel models, as schematized in Fig. 1b, assessing impulsivity, response inhibition, and working memory as mediators of the temporal association of video gaming and attention-deficit/hyperactivity disorder (ADHD) symptoms.

Predictors	Estimate	Std. Er	Pr( >|t|)	95% CI
Video gaming predicting ADHD symptoms through impulsivity				
Intercept (ADHD)	− 3.634	0.202	0.000	− 4.090, − 3.289
Intercept (impulsivity)	10.735	0.195	0.000	10.332, 11.116
Video gaming on ADHD between-person	0.114	0.039	0.000	0.032, 0.191
Video gaming on ADHD within-person	0.063	0.013	0.000	0.038, 0.091
Video gaming on ADHD lagged-within-person	0.024	0.017	0.095	− 0.010, 0.057
Video gaming on impulsivity between-person	0.517	0.046	0.000	0.429, 0.605
Video gaming on impulsivity within-person	0.132	0.019	0.000	0.099, 0.168
Video gaming on impulsivity lagged-within-person	0.044	0.023	0.035	− 0.002, 0.086
Impulsivity on ADHD between-person	0.591	0.014	0.000	0.565, 0.617
Impulsivity on ADHD within-person	0.258	0.007	0.000	0.244, 0.273
Impulsivity on ADHD lagged-within-person	0.031	0.008	0.000	0.014, 0.045
Mediation between-person	**0.306**	**0.028**	**0.000**	**0.255, 0.357**
Mediation within-person	**0.034**	**0.005**	**0.000**	**0.026, 0.044**
Mediation lagged-within-person	0.001	0.001	0.035	0.000, 0.003
Video gaming predicting ADHD symptoms through response inhibition				
Intercept (ADHD)	2.045	0.244	0.000	1.533, 2.450
Intercept (response inhibition)	32.512	1.258	0.000	30.341, 35.196
Video gaming on ADHD between-person	0.378	0.054	0.000	0.278, 0.499
Video gaming on ADHD within-person	0.093	0.017	0.000	0.058, 0.125
Video gaming on ADHD lagged-within-person	0.039	0.020	0.037	− 0.002, 0.073
Video gaming on response inhibition between-person	0.639	0.294	0.007	0.135, 1.210
Video gaming on response inhibition within-person	0.250	0.134	0.030	− 0.011, 0.517
Video gaming on response inhibition lagged-within-person	− 0.001	0.001	0.313	− 0.003, 0.002
Response inhibition on ADHD between-person	0.019	0.004	0.000	0.012, 0.026
Response inhibition on ADHD within-person	0.001	0.001	0.303	− 0.002, 0.003
Response inhibition on ADHD lagged-within-person	− 0.001	0.001	0.313	− 0.003, 0.002
Mediation between-person	**0.012**	**0.006**	**0.007**	**0.002, 0.026**
Mediation within-person	0.000	0.000	0.313	− 0.001, 0.001
Mediation lagged-within-person	0.000	0.000	0.423	− 0.001, 0.001
Video gaming predicting ADHD symptoms through working memory				
Intercept (ADHD)	2.399	0.2014	0.000	2.033, 2.832
Intercept (working memory)	16.812	0.614	0.000	15.482, 17.955
Video gaming on ADHD between-person	0.391	0.044	0.000	0.307, 0.478
Video gaming on ADHD within-person	0.098	0.014	0.000	0.070, 0.126
Video gaming on ADHD lagged-within-person	0.042	0.017	0.003	0.007, 0.079
Video gaming on working memory between-person	0.865	0.158	0.000	0.589, 1.167
Video gaming on working memory within-person	0.053	0.065	0.220	− 0.077, 0.184
Video gaming on working memory lagged-within-person	− 0.096	0.088	0.157	− 0.260, 0.097
Working memory on ADHD between-person	0.017	0.006	0.010	0.006, 0.030
Working memory on ADHD within-person	0.004	0.002	0.057	− 0.001, 0.008
Working memory on ADHD lagged-within-person	0.004	0.002	0.023	0.000, 0.009
Mediation between-person	**0.015**	**0.006**	**0.010**	**0.004, 0.027**
Mediation within-person	0.000	0.000	0.250	0.000, 0.001
Mediation lagged-within-person	0.000	0.001	0.167	− 0.002, 0.000

The significant mediated between- and within-person associations are highlighted in boldface (*p* < 0.050, one-tailed *p*-value).

Findings showed a statistically significant association of social media use to ADHD symptoms mediated by impulsivity at both between-person and concurrent within-person levels, as well as at lagged-within-person level (which translates to a mediated path over 3 years, with the predictor, mediator, and the outcome consecutively measured 1 year apart). With regards to the association of social media to ADHD symptoms mediated by neuropsychological functions, ADHD symptoms were predicted by social media use only through response inhibition at between-person level.

We found a statistically significant impulsivity-mediated association of television viewing on ADHD symptoms at both between-person and concurrent within-person levels, while it showed a statistically significant association with ADHD symptoms mediated by both response inhibition and working memory only at between-person level.

Video gaming revealed a statistically significant impulsivity-mediated association with ADHD at between- and concurrent within-person levels, while response inhibition and working memory revealed a statistically significant mediation role only at between-person level.

### Four-level chained mediation analyses

Considering that impulsivity proved to be a robust mediator of the longitudinal relationship between screen time and ADHD symptoms, MLM models investigated potential chained mediation processes whereby neuropsychological functions were considered as potential intermediate mediators between screen time and impulsivity, which in turn predicted ADHD symptoms 1 year later.

As first step, two different mediation MLMs were performed where impulsivity was predicted by screen time through each of the neuropsychological functions (see the model schematized in Fig. 1c). Results are shown in Table 5. Social media use showed a significant association with impulsivity mediated by response inhibition at between-person and concurrent within-person levels, and by working memory at between-person level. Television viewing and video gaming showed significant associations with impulsivity through response inhibition and working memory only at between-person level.

**Table 5:** Estimated parameters for mediation multilevel models, as schematized in Fig. 1c, assessing the effect of screen time on impulsivity through neuropsychological functions.

Predictors	Mediators							
	Response inhibition				Working memory			
	Estimate	Std. Er	Pr( >|t|)	95% CI	Estimate	Std. Er	Pr( >|t|)	95% CI
Social media use predicting impulsivity through the mediator								
Between-person	**0.041**	**0.014**	**0.000**	**0.016, 0.070**	**0.040**	**0.011**	**0.000**	**0.020, 0.066**
Within-person	**0.002**	**0.001**	**0.005**	0.000, 0.005*	0.001	0.001	0.025	0.000, 0.002
Lagged-within-person	0.000	0.001	0.420	− 0.002, 0.001	0.001	0.001	0.090	0.000, 0.002
Television viewing predicting impulsivity through the mediator								
Between-person	**0.028**	**0.011**	**0.000**	**0.011, 0.053**	**0.026**	**0.010**	**0.000**	**0.009, 0.046**
Within-person	0.000	0.001	0.473	− 0.002, 0.002	0.001	0.001	0.045	0.000, 0.002
Lagged-within-person	0.000	0.001	0.423	− 0.001, 0.001	0.000	0.001	0.385	− 0.002, 0.001
Video gaming predicting impulsivity through the mediator								
Between-person	**0.017**	**0.009**	**0.003**	**0.002, 0.036**	**0.037**	**0.010**	**0.000**	**0.017, 0.056**
Within-person	0.001	0.001	0.073	0.000, 0.003	0.000	0.000	0.260	− 0.001, 0.001
Lagged-within-person	0.000	0.000	0.473	− 0.001, 0.001	0.000	0.001	0.130	− 0.002, 0.000

The significant between- and within-person associations are highlighted in boldface (*p* < 0.050, one-tailed *p*-value). *CI does not include/cross zero.

Two final analyses applying a 4-variable 4-year chained mediation MLMs with multiple mediators of interest were conducted (see the model schematized in Fig. 1d). In these models, ADHD symptoms were predicted by screen time through two chained mediators, i.e., each neuropsychological function and impulsivity. Results are shown in Table 6. Over and above significant between-person relationships, social media use showed significant concurrent within-person effects on ADHD symptoms through response inhibition on impulsivity, while television viewing and video gaming showed only significant between-person effects through response inhibition and working memory.

**Table 6:** Estimated parameters for 4-variable chained mediation multilevel models, as schematized in Fig. 1d, assessing the effect of screen time on attention-deficit/hyperactivity disorder (ADHD) symptoms through multiple chained mediators.

Predictors	Multiple chained mediators							
	Response inhibition on impulsivity				Working memory on impulsivity			
	Estimate	Std. Er	Pr( >|t|)	95% CI	Estimate	Std. Er	Pr( >|t|)	95% CI
Social media use predicting ADHD symptoms through chained mediators								
Between-person	**0.022**	**0.008**	**0.000**	**0.007, 0.038**	**0.024**	**0.007**	**0.000**	**0.011, 0.037**
Within-person	**0.001**	**0.000**	**0.013**	0.000, 0.001*	0.000	0.000	0.130	0.000, 0.001
Lagged-within-person	0.000	0.000	0.485	0.000, 0.000	0.000	0.000	0.120	0.000, 0.000
Television viewing predicting ADHD symptoms through chained mediators								
Between-person	**0.015**	**0.006**	**0.000**	**0.004, 0.027**	**0.015**	**0.006**	**0.005**	**0.003, 0.026**
Within-person	0.000	0.000	0.425	− 0.001, 0.001	0.000	0.000	0.100	0.000, 0.001
Lagged-within-person	0.000	0.000	0.435	0.000, 0.000	0.000	0.000	0.435	0.000, 0.000
Video gaming predicting ADHD symptoms through chained mediators								
Between-person	**0.009**	**0.005**	**0.015**	**0.000, 0.020**	**0.021**	**0.005**	**0.000**	**0.010, 0.033**
Within-person	0.000	0.000	0.070	0.000, 0.001	0.000	0.000	0.250	0.000, 0.000
Lagged-within-person	0.000	0.000	0.490	0.000, 0.000	0.000	0.000	0.290	0.000, 0.000

The significant between- and within-person associations are highlighted in boldface (*p* < 0.050, one-tailed *p*-value). *CI does not include/cross zero.

## Discussion

Using a large population-based sample of nearly 4000 Canadian adolescents to investigate longitudinal direct and indirect associations of different types of screen time (i.e., social media use, television viewing, video gaming, and computer use) with ADHD symptoms, this study distinguished between different time-varying factors: between-person effect, concurrent within-person effect, and lagged-within-person effect. Different potential mediators of the association of screen time and ADHD (i.e., impulsivity, response inhibition, and working memory) were evaluated, and several important conclusions can be drawn.

First, our findings indicated that social media use, television viewing and video gaming were shown to be directly associated with ADHD symptoms during adolescence at both between- and concurrent within-person levels. In other words, the results demonstrated significant common vulnerability between higher levels of these types of screen time and general vulnerability to ADHD symptoms. Over and above this potential common vulnerability, increases in screen time on a given year predicted concurrent increases in ADHD symptoms relative to a person’s mean level of ADHD symptoms throughout adolescence, further suggesting a direct short-term negative impact of screen time on ADHD symptoms.

The study did not reveal a significant mediating role of both neuropsychological functions in the longitudinal association of screen time and ADHD symptoms (indirect lagged within-person effect), but did demonstrate significant concurrent and lagged relationships of social media with ADHD symptoms through impulsivity at within-person level, implying that any further increases in a given year of social media use were associated with more severe ADHD symptoms that same year and from 1 year to the next through higher self-reported impulsivity. This means that social media heavy users showed the strongest effect on ADHD symptoms through impulsive behavior compared with their peers, showing also a mediated effect on their ADHD symptoms on any given year comparing with their average set point, and a small-mediated effect on their ADHD symptoms in the following year.

Trait impulsivity, which moderately correlates with individual neuropsychological measures, like response inhibition and working memory, is considered a multifaceted construct and a key proximal indicator of risk for externalising mental health problems^25,45^. Accordingly, we further investigated the association of screen time and ADHD symptoms, testing the mediational role of neurophysiological functions in the relationship between screen time and this important proximal mediator of risk in a MLM with multiple chained mediators. Results showed that the common vulnerability to greater social media use, television viewing, and video gaming and ADHD behaviors was mediated by neurophysiological functions and impulsivity at between-person levels. However, only social media was shown to be longitudinally linked to increased risk for ADHD symptoms through a mediation path that involved disrupted neuropsychological functioning (inhibitory control) and impulsive temperament.

Altogether, our findings are in line with previous longitudinal studies showing significant associations between digital media use and ADHD symptom severity^40^, and stress the importance of monitoring screen time during adolescence in order to protect those at risk for heavy screen time and to reduce the likelihood of ADHD symptoms being exacerbated through disrupted neurocognitive functioning and behavioural regulation.

According to our results, it appears that there is strong evidence for a common vulnerability to heavy screen time use and adolescents’ neurocognitive risk for ADHD symptoms. It might be argued that adolescents vulnerable to ADHD or those with higher levels of ADHD symptoms are particularly drawn to digital media because they offer activities presenting them with brief, superficial and stimulating parcels of content, without much need to exert cognitive control and allowing for constant attentional switching^46^. Indeed, according to the American Psychiatric Association^47^, adolescents who live with ADHD often have difficulties in finishing cognitive tasks requiring extended periods of sustained attention and show a preference for immediate as opposed to delayed rewards, because of their impulsive attitude. This symptom profile is, in fact, characterized by decreased activation of brain regions implicated in cognitive control and inhibitory control systems^48^. Children with higher polygenic risk score for ADHD present alterations in white matter tracts related to visual functions, resulting in impaired executive control of visual functions, and making subjects with ADHD symptoms more sensitive to external visual stimuli and more easily distracted by digital media^49^. Based on our findings, we posit that social media, television and video games exposure, which showed between-effects on ADHD symptoms mediated by impulsivity and neuropsychological functions, might appeal to adolescents with higher levels of ADHD symptoms.

**Figure 2:**
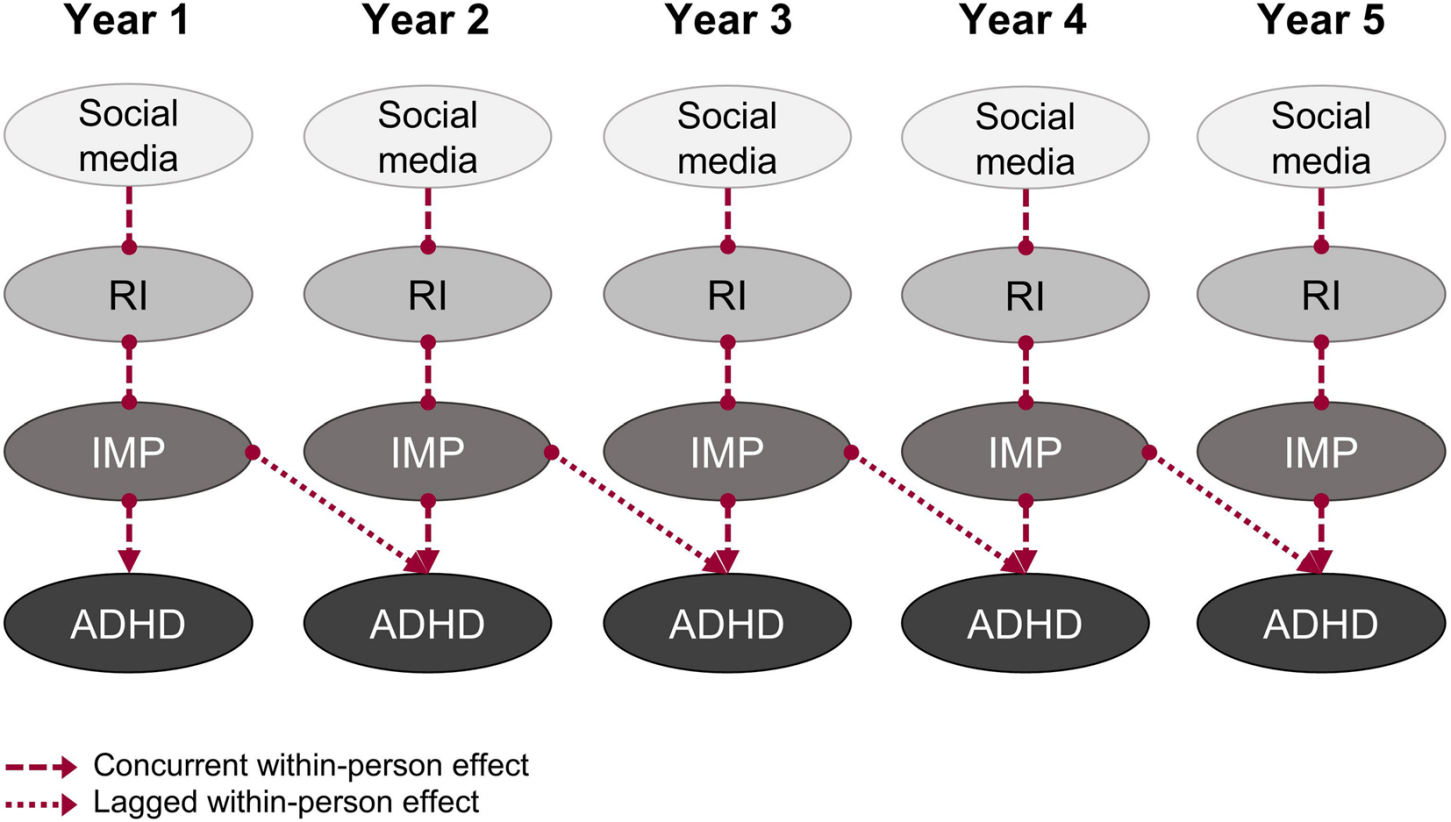
Empirical model of longitudinal neuropsychological functions consequences of social media on behavior in adolescents. ADHD, attention-deficit/hyperactivity disorder symptoms; IMP, impulsivity; RI, response inhibition; Year 1: assessment with first survey wave at 7th grade, and so on.

Over and above this common vulnerability and focusing on the longitudinal risk for ADHD highlighted by this study, our findings support an empirical model showing neuropsychological consequences of digital media use on cognition and temperament during adolescence, where social media proved to be the most robust longitudinal predictor for ADHD symptoms and this relationship was explained by the effects of social media on response inhibition and impulsivity through a chained pathway. Interestingly, in this chained pathway of effects, temporal precedence was demonstrated between social media and impulsivity and between impulsivity and ADHD symptoms, but neuropsychological outcomes of social media only proved to be concurrent. As shown in Fig. 2, increased time spent in front of social media leads to neurocognitive inhibitory control difficulties, which in turn result in a concurrent increase in self-reported impulsive behaviors and a consequent increase in ADHD symptoms in the same year, as well as the next year. These findings were shown to be robust even after accounting for the potential common vulnerability between these behaviors and neurocognitive difficulties, in a manner that is consistent with a causal hypothesis.

The mechanisms by which social media have a unique relationship to impaired response inhibition, are not well understood, but might be related to the nature of the brief and unnarrated content that youth are exposed to on social media. Indeed, social media involves rapidly scrolling through brief, diverse segments of visual information with sound bites, which requires limited cognitive control (e.g., images, brief posts or status updates). Interacting with this content can be characterized by instant rewards and constant stimulation that could raise the threshold for adolescent sustained attention on other less stimulating situations and tasks, exacerbating inattention and distractibility^41,50^. Compared to video game use, social media users’ responses are very limited, defined by a simple gesture in the form of short phrases, likes and/or emoji and are not goal oriented. Similarly, compared to content viewed on television, social media also rarely contains a sustained narrative, involving plot or character development, or a series of facts linked to an argument or a thesis. Finally, social media involves viewing content shared by peers, which, more strongly promotes social norms in young people, for better or worse^51^.

While this study did not focus on the interaction between risk factors and consequences of digital media on mental health, it is noteworthy that youth with ADHD often experience difficulties with behavior, self-regulation, and task focus making them vulnerable to social isolation and other disabilities^52–54^, and high exposure to screen time appears to contribute to some of these symptoms and exacerbate them in those who are already vulnerable^39^. A major strength of our study is the use of state-of-the-art multilevel mediation modeling to investigate the underlying mechanism of this association, confirming the role of impulsivity and executive functions in linking screen time to ADHD symptoms in adolescence. This model provides an overall picture of the onset and development of ADHD symptoms by highlighting specific longitudinal direct and indirect between- and within-person associations, considering concurrent and lagged time-varying effects. Another strength of the study is the use of a large sample size of adolescents.

However, the findings need to be interpreted in light of some limitations. It remains unclear what specific types of social media, digital media content, and what kind of video games or computer-related activities youth participated in. Additionally, even though ADHD symptoms were evaluated with the five items of the Hyperactivity/Inattention scale of the validated and reliable Strength and Difficulties Questionnaire^55^, no clinical diagnostic confirmation was provided and participants may have under-reported their ADHD symptoms due to social desirability or lack of insight into impairment.

In conclusion, in the present study, social media was found to be the type of screen time most robustly associated with symptoms of ADHD in adolescence, while impulsivity seemed to be the strongest mediator between screen time and ADHD symptoms at between- and concurrent within-person levels, as well as at lagged-within-person level. Because of the implications of these findings for interventions, clinicians may want to examine the degree to which adolescents spend time in front of various digital screens and target social media use during therapy as a main potential means of reducing cognitive, behavioral and clinical manifestations of these patterns of difficulties. Furthermore, since the association of all studied types of screen time and ADHD symptoms were mediated by impulsivity, interventions targeting impulsive temperament and behavior and the role of screen time in both managing and exacerbating impulsivity could help young people and their families to better manage ADHD symptoms to promote well-being. Finally, further longitudinal studies are needed to understand the impact of specific contents, and presentation formats on ADHD-related behaviors among adolescents to potentially inform how social media platforms could provide services to families without these potential unintended cognitive and behavioral consequences. Such research will require close collaborations with social media companies to be able to better investigate how the algorithmic nature of content presentation contributes to the potential impact on child brain and mental health.

## Methods

### Participants

This study used data from the Co-Venture cohort, which has an embedded cluster randomized trial for a subset of high-risk youth (for further details see^56^). From September 2012, 3826 adolescents (1798 girls [47%]; mean [SD] age, 12.7 [0.5] years) from 7th grade were recruited with no exclusion criteria from 31 representative schools in the Greater Montreal Area (QC, Canada) with respect to size, socioeconomic indicators, and district. This cohort represents a unique research opportunity in the history of digital media, when teen access to smartphones and social media platforms exploded and adolescent-targeted products and platforms became more and more available^57^. In total, 3779 participants (1858 girls [49%]; mean [SD] age: 12.8 [0.5] years) consented and completed the survey in the 7th grade and were invited to complete repeated assessments every year for five consecutive years until the end of high school (11th grade). Data were acquired through a confidential annual web-based survey, which was downloaded to school computer laboratories to ensure that time-sensitive data (e.g., reaction time) were reliably collected and that students were closely supervised when completing the battery. The scores were saved on computers until a secure connection was available and then automatically transferred to a central anonymized database through a unique identifying code.

This research complies with all relevant ethical guidelines and regulations. Ethical approval was obtained from the Research Ethics Board of the Sainte-Justine Hospital Research Center in Montreal (QC, Canada). Students and their parents or legal guardians provided informed consent before taking part in the study. This study has been registered as sub-study from Co-Venture trial with the number NCT01655615 at http://www.clinicaltrials.gov (date of first trial registration 02/08/2012).

### Measures

#### Screen time

Screen time was assessed using a self-report question requiring participants to state how much time per day they spend using social media (i.e., Facebook, Twitter, or other social networking sites), watching shows or movies on television or on the computer, playing video games (on the computer, cell phone, game console), and using the computer to engage in another kind of activities. The amount of screen time was established according to four categories: 0 to 30 min, 30 min to 1 h and 30 min, 1 h and 30 min to 2 h and 30 min, 2 h and 30 min to 3 h and 30 min, and 3 h and 30 min or more.

#### Attention-deficit/hyperactivity disorder symptoms

ADHD symptoms were assessed using the five items of the Hyperactivity/Inattention scale of the Strength and Difficulties Questionnaire^55^, measuring the key symptoms of ADHD: inattention (two items), hyperactivity (two items) and impulsiveness (one item). Participants indicated whether each statement was not true, somewhat true, or certainly true, yielding a final score from zero to two points.

#### Impulsivity

Participant impulsivity was assessed using five items related to the impulsivity personality dimension of the Substance Use Risk Profile Scale, which consists of twenty-three items that allow identifying four personality dimensions (i.e., impulsivity, anxiety sensitivity, hopelessness, and sensation seeking)^58^. Participants indicated whether they agreed with each statement by selecting one of four response options (strongly disagree, disagree, agree, strongly agree), yielding scores from one to four points.

#### Neuropsychological functions

A modified version of the Go/No-Go Passive Avoidance Learning Paradigm (PALP) was used to measure response inhibition in order to study inhibitory control and cognitive control. The Go/No-Go PALP is a discrimination task that demands participants to inhibit a rewarded response in order to win points or prevent loss of points^59^. During the task, participants learn to react to “good’’ numbers and to avoid reacting to “wrong’’ numbers through trial and error. Correct and incorrect responses were respectively accompanied by reward cues and punishments. Participants received performance feedback following each response. The dependent measure on this task is the total number of errors of commission (responding to no-go numbers).

Working memory was assessed using the “Find the phone task” which is similar to the Self-Order Pointing Task^60^ and the spatial working memory task of the Cambridge Neuropsychological Test Automated Battery^61^. This task presented participants with a number of colored boxes, which required them to remember which boxes they already searched. Participants were required to find a phone ‘token’ hidden amongst the boxes through trial and error. The location and colour of the boxes were changed from trial to trial and with the number of boxes progressively increasing (max. 8) to prevent reliance on stereotyped search strategies. The measure of spatial working memory deficit corresponds to the number of times that participants reselected the items that had already rung.

#### Covariates

We assessed baseline socio-economic status with the Family Affluence Scale for adolescents^62^ and sex (1 = male, 2 = female).

### Statistics

We applied Bayesian MLMs to investigate direct and indirect associations of screen time (independent variable, predictor) and ADHD symptoms (dependent variable, outcome). The MLMs apply linear regression analysis to describe outcome variables as a function of predictor variables at two levels of data organization, the between-person and the within-person (change over time) levels. The between-person effects (the association between the predictor and the outcome variables averaged over five time points), the concurrent within-person effects (the associations between the predictor and the outcome variables in individual participants on a given year), and the lagged-within-person effects (the associations between the predictor on a given year and the outcome variables the following year in individual participants) were assessed within the same MLM at each analytical step. To assess time-varying associations, significant within-person associations would provide support for potential causal short-term concurrent associations between variables, while significant lagged-within-person associations would reflect a lasting effect of screen time on adolescent behavior.

Figure S1 in Supplementary Information provides visual representation of observed and derived (latent) variables for an analysis of relationships between two sets of variables. Covariates (sex, socio-economic status) were included at the between level. Because mediators were also measured at every time point, they were tested at between and within level. MLMs also provide estimates of the indirect effects of the predictor through the mediator or a chained set of mediators (see Supplementary Figs. S2 and S3, respectively) that, with the use of the lagged effect, can be temporally specified.

We first tested the direct association of screen time and ADHD symptoms by entering the four types of screen time in the same MLM (Fig. 1a). Second, we ran different independent MLMs to assess the role of impulsivity and neuropsychological functions (i.e. response inhibition and working memory) as mediators of an indirect 3-year relationship between relevant types of screen time and ADHD symptoms (Fig. 1b). Mediation models were only performed for screen time variables that showed significant relationships to ADHD symptoms.

To further characterize mediation effects in the context of multiple mediation variables, we also conducted independent mediation MLMs to analyze the associations between relevant types of screen time and impulsivity through both neuropsychological functions (Fig. 1c), with the rationale that changes in cognition would first lead to changes in a core symptoms of ADHD, which, over time would contribute to the development of a larger spectrum of ADHD symptoms^19^. In the context of such a mediation, we then tested 4-variable chained mediation MLMs, where the multiple sequential mediators were either response inhibition and impulsivity, working memory and impulsivity (Fig. 1d).

All analyses were performed using Mplus software (version 8.3, Muthén & Muthén, Los Angeles, CA). In all statistical models, we controlled for covariates (i.e., sex, socio-economic status) and time. The time parameter of our 5-year study was coded from one to five, considering the five survey waves as time points. To model the indirect effects of screen time on ADHD symptoms, the “model constraint” function in Mplus allowed multiplying the direct associations between predictors, mediators and outcomes.

### Supplementary Information

Supplementary Information.

## Data Availability

A summary of the data that support the findings of this study is available from the corresponding author on reasonable request.
