# Supplementary material for: Screen time, impulsivity, neuropsychological functions and their relationship to growth in adolescent attention-deficit/hyperactivity disorder symptoms
Source: Sci Rep. 2023 Oct 23;13:18108. doi: 10.1038/s41598-023-44105-7 (PMC10593930; doi:10.1038/s41598-023-44105-7)

Screen time, impulsivity, neuropsychological functions and their relationship to growth in adolescent Attention-Deficit/Hyperactivity Disorder symptoms

Jasmina Wallace[1, 2*], Elroy Boers[1, 2], Julien Ouellet[1, 2], Mohammad H. Afzali[1, 2], Patricia Conrod[1, 2]

[1]CHU Sainte-Justine Research Center, Montreal, Montreal, QC, Canada

[2]Department of Psychiatry, University of Montreal, Montreal, QC, Canada

SUPPLEMENTARY INFORMATION

Table S1. Descriptive statistics across the five survey waves.

	Year 1		Year 2		Year 3		Year 4		Year 5	
	M	SD	M	SD	M	SD	M	SD	M	SD
**Age (years)**	12.80	0.50	13.80	0.40	14.70	0.50	15.80	0.40	16.80	0.40
**Social media use (min)**	42	63	50	67	55	66	63	66	70	66
**Television viewing (min)**	66	63	68	64	71	65	72	66	72	66
**Video gaming (min)**	59	69	65	73	65	74	62	75	55	72
**Computer use (min)**	25	50	25	50	24	48	26	51	26	50
**ADHD symptoms**	3.93	2.31	4.06	2.35	4.12	2.41	4.12	2.38	4.06	2.36
**Impulsivity**	11.88	2.84	11.74	2.87	11.67	2.93	11.54	2.87	11.31	2.84
**Response inhibition errors**	31.28	17.62	25.49	17.56	21.09	16.05	18.27	15.46	16.16	15.02
**Working memory errors**	16.05	10.19	13.53	9.77	10.90	8.91	9.74	8.65	8.89	8.40

Note. Min, minutes; Year 1: assessment with first survey wave at 7th grade, and so on.

Table S2. Frequency distribution for different forms of screen time over five survey waves.

	0 to 30 min		30 min to 1h and 30 min		1h and 30 min to 2h and 30 min		2h and 30 min to 3h and 30 min		3h and 30 min or more		Missing	
	N	%	N	%	N	%	N	%	N	%	N	%
**Social media use**												
Year 1	1979	52.40	843	22.30	436	11.55	217	5.70	293	7.75	11	0.30
Year 2	1385	36.65	828	21.90	443	11.70	243	6.45	290	7.70	590	15.60
Year 3	1054	27.90	877	23.20	493	13.00	233	6.20	263	6.90	859	22.80
Year 4	734	19.40	930	24.60	552	14.60	286	7.60	270	7.15	1007	26.65
Year 5	531	14.00	886	23.50	578	15.30	302	8.00	270	7.20	1212	32.00
**Television viewing**												
Year 1	704	18.65	1441	38.10	923	24.45	363	9.60	337	8.90	11	0.30
Year 2	598	15.85	1194	31.60	748	19.80	341	9.00	308	8.15	590	15.60
Year 3	540	14.30	1025	27.10	722	19.00	343	9.10	290	7.70	859	22.80
Year 4	578	15.30	864	22.85	706	18.70	337	8.90	287	7.60	1007	26.65
Year 5	510	13.50	837	22.15	625	16.55	336	8.90	259	6.90	1212	32.00
**Video gaming**												
Year 1	1220	32.30	1210	32.00	633	16.70	272	7.20	433	11.50	11	0.30
Year 2	1038	27.50	917	24.30	512	13.50	281	7.40	441	11.70	590	15.60
Year 3	1041	27.55	720	19.00	488	12.90	265	7.00	406	10.75	859	22.80
Year 4	1127	29.80	591	15.65	425	11.30	242	6.40	387	10.20	1007	26.65
Year 5	1157	30.60	554	14.70	362	9.60	179	4.70	315	8.40	1212	32.00
**Computer use**												
Year 1	2471	65.40	756	20.00	277	7.30	117	3.10	147	3.90	11	0.30
Year 2	2090	55.30	659	17.50	219	5.80	91	2.40	130	3.40	590	15.60
Year 3	1935	51.20	586	15.50	210	5.60	89	2.30	100	2.60	859	22.80
Year 4	1803	47.70	552	14.65	211	5.60	91	2.40	115	3.00	1007	26.65
Year 5	1629	43.10	550	14.60	206	5.50	92	2.40	90	2.40	1212	32.00

Note. Year 1: assessment with first survey wave at 7th grade, and so on.

Figure S1. Multilevel model with two variables, each observed over five-time points and their derived between-, within- and lagged- latent variables. Rectangles represent observed variables and ovals represent latent variables.

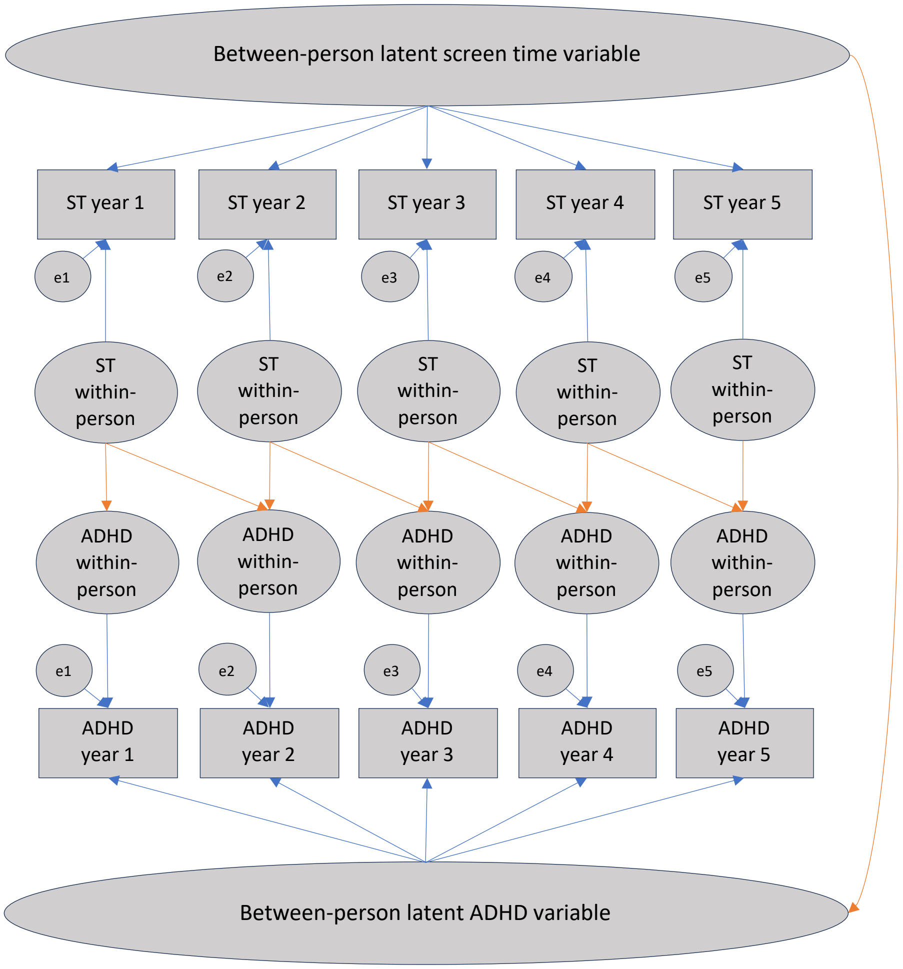

Figure S2. Multilevel model with three variables, each observed over five-time points and their derived between-, within- and lagged- latent variables. Rectangles represent observed variables and ovals represent latent variables.

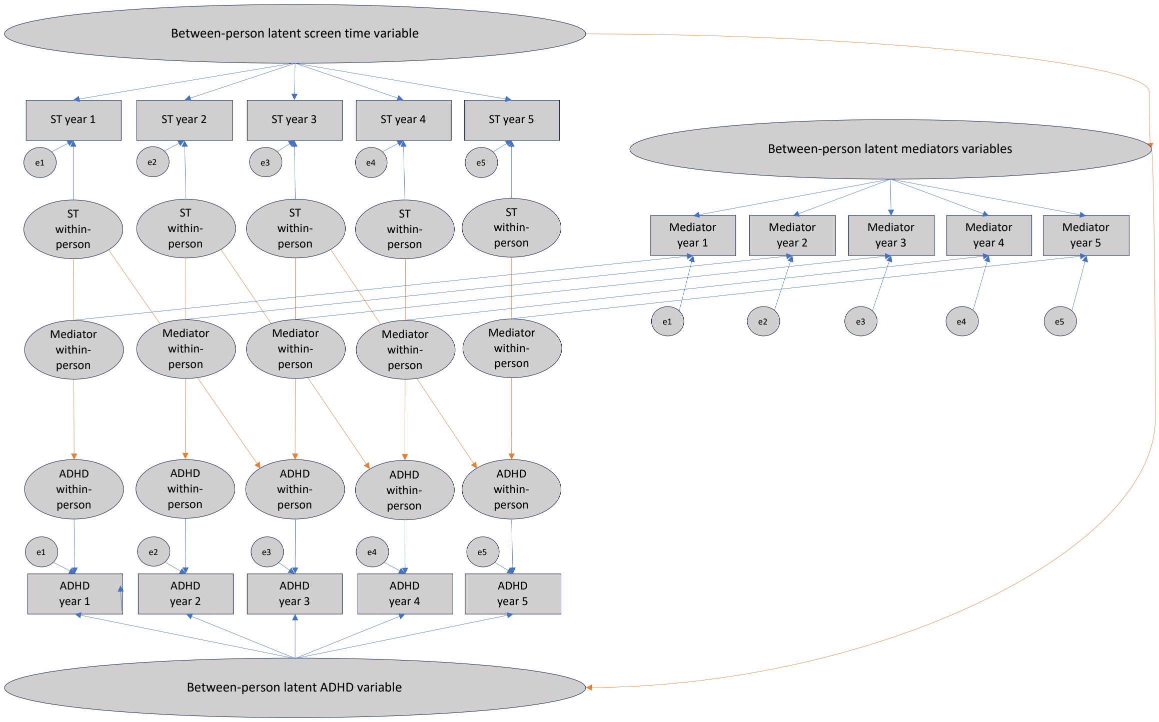

Figure S3. Multilevel model with four chained variables, each observed over five-time points and their derived between-, within- and lagged- latent variables. Rectangles represent observed variables and ovals represent latent variables.

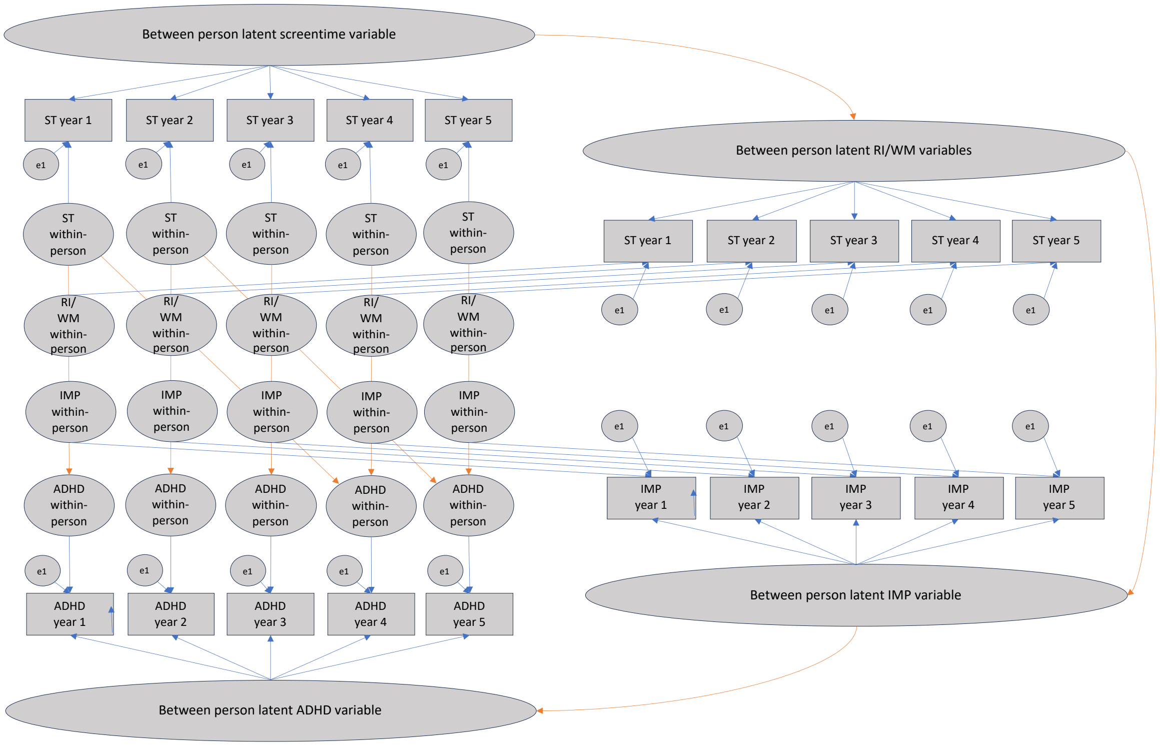

Supplement: Supplementary file 1 — Supplementary Information. [file 41598_2023_44105_MOESM1_ESM.pdf]
